# Comparative Analysis of Volatile Flavor Compounds in Seven Mustard Pastes via HS-SPME–GC–MS

**DOI:** 10.3390/molecules28145482

**Published:** 2023-07-18

**Authors:** Miao Liang, Rui Wang, Yajian Wu, Runhu Xin, Wei Guan, Yuping Liu

**Affiliations:** 1 China Food Flavor and Nutrition Health Innovation Center, Beijing Technology and Business University, Beijing 100048, China; liang_miao923@163.com (M.L.); wangruicoke@163.com (R.W.); 2130031020@st.btbu.edu.cn (Y.W.); 17861506332@163.com (R.X.); 2250031001@st.btbu.edu.cn (W.G.); 2 School of Light Industry, Beijing Technology & Business University, Beijing 100048, China

**Keywords:** mustard paste, headspace solid-phase microextraction, volatile compounds, gas chromatography–mass spectrometry, isothiocyanate, allyl thiocyanate

## Abstract

To identify the volatile flavor components in mustard paste (MP), the volatile compounds in seven MPs available on the market were isolated and analyzed by headspace solid-phase microextraction combined with gas chromatography–mass spectrometry. A total of 27 volatile constituents were found by mass spectra and retention index compared to the data obtained from reference compounds or the related literature and databases; these compounds included nine esters, three sulfur-containing compounds, two nitriles, three ketones, three alkenes, and seven other compounds. Of the 27 compounds, 6 compounds came from the turmeric added to MPs. Among the components detected, some compounds derived from AITC were allyl thiocyanate, carbon disulfide, allyl mercaptan, diallyl sulfide, and diallyl disulfide. The results obtained provide a better and comprehensive recognition of the volatile flavor compounds in MPs, and have some reference values for developing and applying isothiocyanate compounds.

## 1. Introduction

In the 1980s and 1990s, the popularity of raw seafood such as lobster, tuna, and salmon, as well as sushi in China, led to the gradual recognition, acceptance, and enjoyment of mustard paste (MP) as condiments among diners, owing to its characteristic flavor and bactericidal functions. MPs are divided into different grades based on their ingredients used; top-grade mustard sauce is only made from horseradish, middle-grade mustard sauce from horseradish and fine mustard power, and the low-grade contains mustard powder or mustard powder mixed with dabs of horseradish. All MP products possess a strong pungent and stimulating aroma, derived from isothiocyanate (ITC) flavor compounds, which have various antibacterial effects, so these products do not include preservatives [1,2,3]. With the increase in demand and industrial output of these products, the raw materials now employed in MP deviate substantially from those utilized in the traditional method. Moreover, some products contain flavor compounds and food flavorings.

It is reported that allyl isothiocyanate (AITC) is the most important aroma compound in MP products, but, due to its own structural characteristics, AITC is unstable and susceptible to isomerization and degradation [4]. Nowadays, the production of MP involves complex raw materials and diverse processes, while the aroma composition of MP products produced with new raw materials is rarely reported in the published literature, and there are only few reports on the volatiles in MP products. The volatiles in MP were extracted by steam distillation or organic solvent extraction, and separated and identified by gas chromatography (GC) in earlier studies; subsequently, simultaneous distillation extraction [5], headspace solid-phase microextraction (HS-SPME) [6,7], and Soxhlet extraction [8] had primarily been used to isolate aroma components, which were then analyzed using gas chromatography–mass spectrometry (GC–MS) [9]. In addition, most of the volatiles were characterized tentatively by mass spectrometry only, and the relative content was given solely through a normalization method.

The current study aimed to isolate, identify, and compare the volatile compounds present in commercially available MP products and investigate the ITC flavor compounds and their decomposition products. Therefore, the volatiles in seven commercially available MP products were extracted and identified by HS-SPME combined with GC–MS. The obtained results have the potential to provide a theoretical foundation for developing the flavor compounds and food flavorings with mustard notes.

## 2. Results and Discussion

### 2.1. Determination of Emulsion Type of MP

MP is a non-homogeneous system composed of water and oil. To determine the emulsion type of MP, staining method and dilution method were used. The results indicated that MP exhibited characteristics of an oil-in-water emulsion because MP displayed minimal staining when exposed to oil red, but was readily diluted by water and kept its emulsion state.

### 2.2. Comparison of Sensory Evaluation

**Figure 1:**
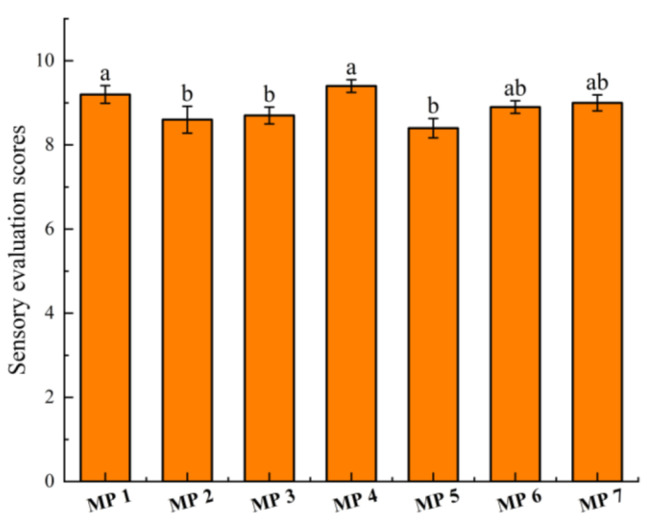
The sensory evaluation scores of seven MPs. Values with different superscript roman letters (a,b) in the column are significantly different according to the Duncan test (*p* < 0.05).

Because the aroma of MP could not be effectively released in paste form, MP was diluted into a 1% aqueous solution to perform the sensory evaluation. The sensory evaluation results of seven MP samples are shown in Figure 1. From the obtained results, it could be seen that MP 4 had the highest score due to its distinct aroma intensity of mustard, which could be attributed to the used materials, including wasabi and fresh wasabi. Meanwhile, MP 5 received the lowest score despite having a pungent aroma, whereas the remaining samples received similar sensory evaluation scores.

### 2.3. Identification of Volatiles in MPs

**Figure 2:**
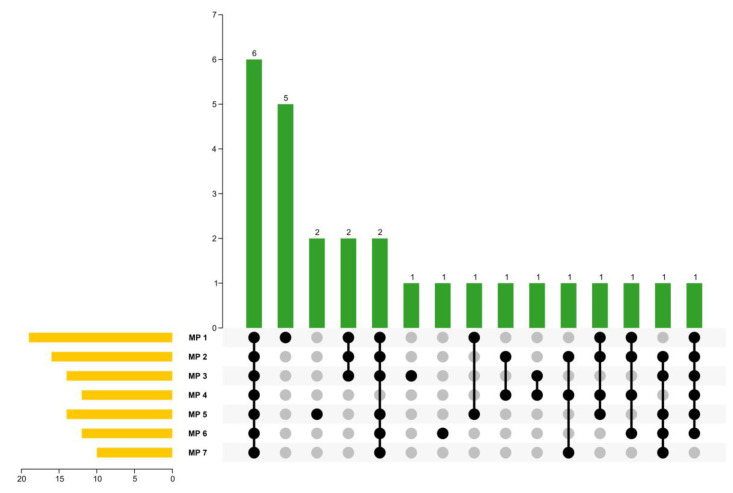
The upset plot of the seven MPs. Yellow bars represent the number of volatile compounds identified in the seven MPs. Gray dots represent absence, while black dots represent the presence of the unique compounds. If more than one sample contains the same unique compound, they are connected with lines. The number of unique compounds presented individually or jointly is represented by green bars.

As shown in Table 1, a total of 27 volatiles were isolated and detected from 7 MP samples, with 19, 16, 14, 12, 14, 12, and 10 compounds in MPs 1, 2, 3, 4, 5, 6, and 7, respectively. Among the 27 volatiles isolated, 12 compounds were positively identified and 15 compounds were tentatively identified. In addition, out of 27 compounds, 17 volatiles had matching degrees greater than 900, indicating that these compounds had excellent matching with the database. Figure 2 indicates that six components were common to all seven samples, including AITC, phenethyl isothiocyanate, allyl thiocyanate, benzenepropanenitrile, carbon disulfide, and allyl chloride. In addition, five compounds were unique to MP 1, including *α*-turmerone, *β*-turmerone, ar-turmerone, (−)-*β*-sesquiphellandrene, and curcumene. The number of volatiles detected in the seven MPs were more than those previously reported in the literature [5]. This could be attributed to the use of a 2 cm-long extractive fiber composed of three materials, which had a better extraction efficiency for the volatiles with different polarities. Furthermore, the raw materials of MP samples used in this experiment might be different from those used in previous studies.

Nine esters were detected in the seven MP samples, the main ones of which were ITCs, which was consistent with the results reported previously [10]. Additionally, three sulfur-containing compounds, three ketones, three alkenes, two nitriles, and seven other compounds were detected in the samples.

Among the 27 volatile components found in the 7 MPs, 3 ketones (*α*-turmerone, *β*-turmerone, and ar-turmerone) and 3 alkenes (*α*-zingiberene, (−)-*β*-sesquiphellandrene, and curcumene) were detected for the first time, which might be related to the turmeric used in the samples. Since turmeric contains the natural pigment curcumin, the mixture of turmeric, lemon yellow, and bright blue pigment could yield the light green color of MP. A previous study reported that six compounds mentioned above (three ketones and three alkenes) were the volatiles of turmeric oil [11], and the use of turmeric in MP could not only play a role in the color but also might have had a certain contribution to the overall aroma profile. Turmeric was used in the MP in samples 1, 2, 3, and 5 as a raw material (Table 2), and six turmeric components mentioned above were all detected in MP 1; only *α*-zingiberene was found in MP 5. However, the turmeric components were not detected in MP 2 and MP 3, possibly due to the low amount of turmeric used in these samples, which primarily served as a toning agent.

AITC, commonly referred to as artificial mustard oil, exhibits a strong characteristic aroma similar to that of mustard. It is produced by the degradation of black mustard glucoside (sinigrin, a kind of thioglucoside) catalyzed by the mustard enzyme [12], and has a threshold value of 0.046 mg/kg in water [13]. AITC plays a crucial role in the aroma of MP products, and it might have originated from various raw materials used in MP, such as mustard oil, wasabi, horseradish powder, fresh wasabi, edible spices, and flavor compounds. Of the seven MPs analyzed, six samples contained horseradish or horseradish powder as a raw material, while mustard oil was used in MP 1. Horseradish powder contained 0.85% volatile oil, which comprised 31.8% AITC [14]. Therefore, the amount of AITC provided by horseradish or horseradish powder was limited, and most of the AITC in MP was likely from the added flavor compounds or food flavoring.

**Figure 3:**
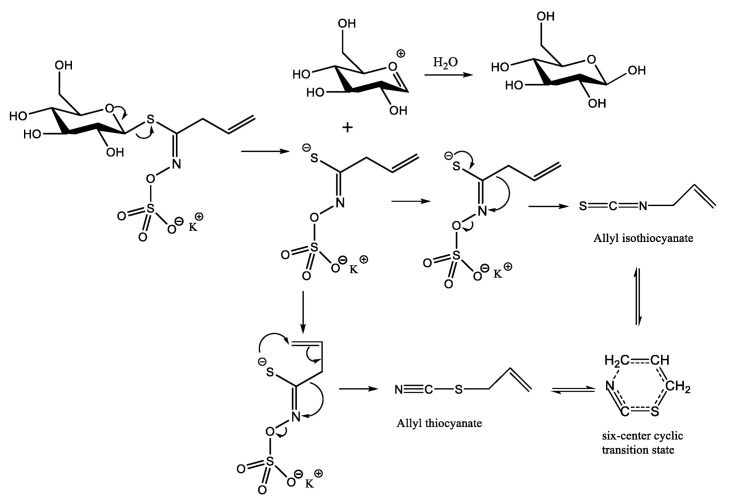
Formation mechanisms of allyl isothiocyanate and allyl thiocyanate.

Both allyl thiocyanate and AITC are isomers (C_4_H_5_NS) with different functional groups. Allyl thiocyanate has two main formation pathways, as depicted in Figure 3. One is that it can be produced by the degradation of black mustard glucoside in the presence of mustard enzyme [15], while the other pathway involves the rearrangement of AITC via a six-membered ring transition state [16]. The peak area ratio of allyl thiocyanate to AITC in the seven MPs ranged from 1:4 to 1:5. Elsewhere, it was reported that the level of allyl thiocyanate in mustard oil was also the second-highest, and the peak area ratio of allyl thiocyanate to AITC was between 1:5.5 and 1:6.5 [17]. It is noteworthy that allyl thiocyanate is currently not a permitted food flavor compound; therefore, there is a need to conduct an in-depth study on controlling its content.

Another compound found among the seven MPs, 3-butenyl isothiocyanate, has a mustard-like pungent aroma and was detected in MP1, MP2, MP4, and MP6 [18]. Phenethyl isothiocyanate had a fresh radish-like pungent note and was identified in all samples; it was noteworthy that the lowest variety of raw materials was used in MP 7, and only AITC and phenethyl isothiocyanate were identified.

In addition, carbon disulfide should be produced by the degradation of AITC [16]. Allyl chloride was identified in all seven MPs, and its source might be associated with the use of AITC. Allyl chloride and sodium thiocyanate were used as raw materials in the synthesis of AITC. At present, both Chinese mandatory standard GB 29980-2013 [19] and the quality specifications of the Joint Expert Committee on Food Additives (JECFA) required that the content of AITC should exceed 98% as a flavor compound. Therefore, allyl chloride might be introduced by adding AITC, which further indicates that AITC might be added in all seven MPs.

Dehydroacetic acid was only detected in MP 6, which is a permitted preservative listed in the Chinese Food Additive Use Standard GB 2760-2014 [20]. However, ITC flavor compounds have antimicrobial and bacteriostatic properties [21], and preservatives are not required during MP production, so dehydroacetic acid detected might be from other raw materials used in the production of MP 6. Other than MP 4, 3-butenenitrile was detected in all samples. The precursors of both nitrile and ITC are thioglucosides, which can be readily degraded to ITCs under neutral conditions and to nitrile under acidic conditions. It was reported that the precursors of 3-butenenitrile and benzenepropanenitrile are sinigrin and gluconasturtiin, respectively [22].

To determine the contribution of volatiles identified to the overall odor profile of MPs, OAVs were calculated according to their concentrations and thresholds in water [13], as shown in Table S2. The odorants with OAV ≥ 1 were determined as the potent odorants of MPs, the number of which were 9, 11, 10, 9, 8, 7, and 6 in the 7 MPs, respectively. The odorant contributing greatly to the aroma of MP was AITC, followed by carbon disulfide, which was a degradation product of AITC which also affects the aroma quality of MPs. Although the potent odorants varied among samples, the OAV of AITC and carbon disulfide were much higher than those of the other aroma components, making the overall aroma of different MP samples similar.

### 2.4. Degradation Products of AITC and Method for Stabilizing AITC

According to the qualitative results, AITC was determined as the primary characteristic odorant in MPs; it could be produced by the degradation of sinigrin or introduced by adding flavor compounds or food flavorings. AITC is known to undergo various chemical reactions, such as hydrolysis, oxidation, thermal degradation, etc., resulting in the formation of hydrogen sulfide, allyl mercaptan, diallyl monosulfide, diallyl disulfide, carbon disulfide, allyl thiocyanate, diallyl thiourea, etc. [16,23], many of which were also detected in this study, leading to variations in the aroma of MP products. There are two approaches that can be employed to avoid unwanted reactions of AITC. The first one is to adjust the pH value of the MP products. ITCs are more stable under acidic conditions, and keeping the pH value of MP products in the acidic range can delay the changes of ITCs to some extent. Citric acid was used in five (MP 1, MP 2, MP 3, MP 4, MP 6) of the seven samples analyzed, and MP 5 contained sodium diacetate. However, acidic conditions are also favorable for the degradation of thioglucosides to nitrile in the raw materials used. Hence, it is crucial to choose a suitable pH value. The second approach involves encapsulating AITC in microcapsules or nanoemulsions using encapsulation techniques, which can also stabilize AITC [24,25].

In addition to the above two methods mentioned, AITC could be replaced by other ITCs, which are more stable and have similar odor characteristics to AITC. More than 200 thioglucosides have been found in plants [26], which means that over 200 ITCs could be formed. From the point of view of flavor chemistry, most of the ITCs exhibited mustard-like characteristics, so structurally stable aroma compounds with mustard characteristics can be designed and synthesized based on the relationship between the molecular backbone and the odor characteristics in combination with the structure of detected thioglucosides. Other than AITC, fourteen types of ITC compounds were approved for use as flavor compounds in Chinese food additive use standard GB2760-2014, including six saturated ITCs (isopropyl isothiocyanate, butyl isothiocyanate, isobutyl isothiocyanate, 2-butyl isothiocyanate, pentyl isothiocyanate, isopentyl isothiocyanate), three unsaturated ITCs (3-butenyl isothiocyanate, 4-pentenyl isothiocyanate, 5-hexenyl isothiocyanate), two aromatic ITCs (benzyl isothiocyanate, phenethyl isothiocyanate), and three saturated ITCs containing methylthio groups (3-(methylthio)propyl isothiocyanate, 5-(methylthio)pentyl isothiocyanate, 6-(methylthio)hexyl isothiocyanate). Most of them have been synthesized; their odor characteristics were evaluated, and the results showed that they had mustardy, pungent, and irritating aromas [27]. Among the synthesized compounds, isopropyl isothiocyanate has almost the same aroma characteristics as AITC, and it is more stable. However, from the volatiles detected in the seven MPs in this study, it could be seen that the above flavor compounds were not as common as AITC. Therefore, in order to improve the aroma quality of MP products, from the point of view of flavor preparation, part or all of AITC should be substituted by other ITCs which have been approved by the Flavor and Extract Manufacturers Association.

## 3. Materials and Methods

### 3.1. Chemicals

A mixture of C_7_–C_30_ normal alkanes in *n*-hexane (HPLC grade) was purchased from Beijing Huawei Ruike Chemical Co., Ltd. (Beijing, China); 6-undecanone (AR) was obtained from Tokyo Huacheng Industrial Pearl Club; sec-butylisothiocyanate-(96%), allyl isothiocyanate (95%), 3-(methylthio)propyl isothiocyanate(98%), benzyl isothiocyanate(97%), phenylethyl isothiocyanate(96%), allyl mercaptan(97%), diallyl sulfide(95%), diallyl disulphide(98%), carbon disulfide(97%), allyl chloride(96%), hexanoic acid(95%), 3-methylphenol(98%), and propylene glycol (99%) were purchased from Sigma-Aldrich Chemical Co. (Saint Louis, USA). Sudan Ⅲ and liquid paraffin were obtained from Beijing Chemical Reagent Co., Ltd. (Beijing, China).

### 3.2. Samples

Seven different MP (43 g) samples were purchased in Beijing Meilianmei Supermarket—Zengguang Road Store. Detailed information is shown in Table 2.

### 3.3. Determination of MP Emulsion Type

Staining method: First, 0.5 g Sudan Ⅲ was dissolved into 100 mL liquid paraffin to obtain oil red solution; then, 1.0 g MP and 1 drop of oil red were put on the glass plate. The mixture was stirred with a glass rod, and its color change was observed.

Dilution method: First, 1.0 g MP and 2 mL deionized water were put on the glass plate. The mixture was stirred with a glass rod, and the change in MP was observed.

### 3.4. Sensory Evaluation of MP

Each MP sample was diluted into a 1% aqueous solution with redistilled purified water, and 10 mL aqueous solution was transferred into a 20 mL odorless glass bottle with a lid. Subsequently, 12 trained sensory evaluators were recruited from the Beijing Key Laboratory of Flavor Chemistry and participated in weekly sensory sessions to train them in recognizing and describing different aroma attributes in order to assess the overall aroma profile of the 7 MP samples. The training lasted for 5−6 weeks, based on their familiarity with the overall aroma profile of MP.

The evaluators employed a 10-point interval scale to rate the samples based on the following criteria: stronger pungent, typical mustard aroma with full aroma (9–10 points, 50 μg/g AITC propylene glycol solution); strong pungent, typical mustard aroma (8–9 points, 40 μg/g); pungent mustard aroma (6–8 points, 30 μg/g); pungent aroma without mustard characteristic (1–6 points, 20 μg/g). The highest and lowest scores were discarded, and the mean of the remaining scores was calculated.

### 3.5. Extraction of Volatiles in MP

The volatiles in MP were extracted by HS-SPME. Accurately 3.0 g MP was measured and put into a clean, odorless bottle (25 mL) with a stir bar. Later, 100 μL 6-undecanone (0.096 μg/L) was added as internal standard. The extraction fiber (DVB/CAR/PDMS, 50/30 μm, 2 cm) used in the experiment was aged at 250 °C in the sample inlet of GC according to the manufacturer’s suggestions. The headspace bottle was sealed and incubated at 45 °C for 20 min, and then the aged fiber was pushed into the headspace bottle and extracted for 50 min at 45 °C. After the extraction, the fiber was injected into the GC injector and desorbed at 250 °C for 5 min for analysis. Analyses of volatile components were conducted in triplicate.

### 3.6. GC–MS Analysis

The volatiles were isolated by GC (Agilent model 7890B) coupled with an Agilent 5975 mass spectrometer detector (MSD) on DB-WAX (30 m × 0.25 mm, 0.25 μm) with helium (≥99.999% purity) at a flow rate of 1.0 mL/min as a carrier gas; the split ratio was 15:1. The initial oven temperature was 50 °C, held for 2 min; it was then raised to 110 °C at the rate of 10 °C/min and held for 5 min; it was subsequently ramped at a rate of 10 °C/min to 150 °C (held for 2 min); finally, it was raised at a rate of 15 °C/min to 240 °C, at which the holding time was 5 min. Electron ionization (70 eV) and full scan mode were used for detection; the range of m/z was 35 to 350. The quadrupole and ion source temperatures were 150 °C and 230 °C, respectively.

### 3.7. Qualitative Analyses

The compounds were tentatively identified by matching their mass fragmentation patterns with the mass spectrometry library (NIST 2014) and retention index (RI); if the standard compounds were available, the compounds were identified positively by comparing their mass spectrum data and RI with those of standard compounds. The volatiles were quantitated simply by an internal standard method (the results obtained are presented in Table S1); their odor activity values (OAVs) were calculated and are presented in Table S2.

### 3.8. Statistical Analysis

Diagrams were drawn with origin 2021b, Microsoft Excel 2021, and TB tools.

## 4. Conclusions

HS-SPME combined with GC–MS was used to isolate and detect the volatiles in seven commercially available MP samples. Twenty-seven compounds were found, among which esters (predominantly ITCs) accounted for the majority. The total number of components detected exceeded the number reported in the literature. Additionally, six compounds derived from turmeric, including α-turmerone, *β*-tumerone, ar-turmerone, α-zingiberene, (−)-*β*-sesquiterpene, and curcumene, were detected for the first time in MPs. Notably, except for AITC, ITC flavor compounds have not been used in MP. Thus, the development and application of ITC compounds deserves to be studied in depth.

## Figures and Tables

**Table 1:** The volatile compounds detected in seven MP samples.

Compounds	Concentration (μg/g)							Matching Degree		RI/RI *^c^	Qualitative Method ^e^
	MP 1	MP 2	MP 3	MP 4	MP 5	MP 6	MP 7	Match	R.Match		
***Esters (9)***											
Allyl isocyanate	+ ^a^	+	+	− ^b^	+	+	+	909	917	972/N ^d^	MS
sec-Butylisothiocyanate-	+	+	−	+	+	−	−	868	895	1289/1287	MS, RI, S
Allyl isothiocyanate	+	+	+	+	+	+	+	960	960	1363/1361	MS, RI, S
Allyl thiocyanate	+	+	+	+	+	+	+	878	910	1447/1440	MS, RI
3-Butenyl isothiocyanate	+	+	−	+	−	+	−	789	803	1451/1453	MS, RI
Diglycolmonoethylether acetate	+	+	+	−	−	−	−	886	901	1684/N	MS
3-(Methylthio)propyl isothiocyanate	−	+	−	+	−	−	−	901	912	1938/1979	MS, S
Benzyl isothiocyanate	+	+	+	+	+	+	−	854	883	2097/2107	MS, RI, S
Phenylethyl isothiocyanate	+	+	+	+	+	+	+	948	948	2227/2234	MS, RI, S
***Sulfur-containing compounds (3)***											
Allyl mercaptan	+	+	+	−	−	−	−	942	946	886/887	MS, RI, S
Diallyl sulfide	−	+	+	−	+	+	+	925	925	1148/1148	MS, RI, S
Diallyl disulphide	−	−	−	−	+	−	−	910	932	1477/1475	MS, RI, S
***Nitriles (2)***											
3-Butenenitrile	+	+	+	−	+	+	+	977	977	1176/1186	MS, RI
Benzenepropanenitrile	+	+	+	+	+	+	+	936	936	2035/2041	MS, RI
***Ketones (3)***											
*α*-Turmerone	+	−	−	−	−	−	−	825	838	2183/2245	MS
*β*-Turmerone	+	−	−	−	−	−	−	850	867	2250/N	MS
ar-Turmerone	+	−	−	−	−	−	−	845	880	2262/N	MS
***Alkenes (3)***											
*α*-Zingiberene	+	−	−	−	+	−	−	861	892	1718/1715	MS, RI
(−)-*β*-Sesquiphellandrene	+	−	−	−	−	−	−	905	919	1765/1765	MS, RI
Curcumene	+	−	−	−	−	−	−	913	965	1769/1768	MS, RI
***Others (7)***											
Carbon disulfide	+	+	+	+	+	+	+	930	960	730/733	MS, RI, S
Allyl chloride	+	+	+	+	+	+	+	866	893	806/814	MS, RI, S
Isothiazole	−	−	+	+	−	−	−	899	908	1214/N	MS
*N*-Allylacetamide	−	−	−	−	+	−	−	831	852	1761/N	MS
Hexanoic acid	−	+	−	+	−	−	+	949	952	1850/1850	MS, RI, S
3-Methylphenol	−	−	+	−	−	−	−	936	945	2087/2085	MS, RI, S
Dehydroacetic acid	−	−	−	−	−	+	−	920	921	2374/N	MS

^a^ “+”: Detected; ^b^ “−”: Not detected; ^c^ RI: retention index on a DB-WAX column, RI *: retention index in the literature (https://webbook.nist.gov/chemistry/ (accessed on 20 March 2023)); ^d^ “N”: retention index was not found in the literature; ^e^ “MS” means identified by mass spectrometry, “RI” means identified by retention index, and “S” means identified by the standard compounds.

**Table 2:** The materials used in the seven MP samples.

Sample	Common Raw Materials	Flavor Material	Other Raw Materials
MP 1	Water; edible oil; sorbitol; salt; tartrazine; brilliant blue (pigment)	Mustard oil	Lactose, refined cane sugar, citric acid, corn starch, xanthan gum, turmeric
MP 2		Horseradish, edible spice	Lactose, citric acid, glyceryl monostearate, xanthan gum, turmeric
MP 3		Horseradish, edible spice	Lactose, citric acid, glyceryl monostearate, avicel, xanthan gum, turmeric
MP 4		Wasabi, fresh wasabi	Glucose, citric acid
MP 5		Horseradish, spice, flavoring essence	Lactose, ansemi, xanthan gum, sodium diacetate, turmeric
MP 6		Horseradish powder	Citric acid, edible starch
MP 7		Horseradish powder	No other ingredient

## Data Availability

The data are available upon reasonable request.

## Supplementary Materials

The following supporting information can be downloaded at: https://www.mdpi.com/article/10.3390/molecules28145482/s1, Table S1: The relative concentration of volatile compounds in seven MP samples; Table S2: Odor activity values of volatiles in seven samples.

## Sample Availability

Samples of the compounds are not available from the authors.

## References

[1] Nielsen P.V., Rios R. (2000). Inhibition of fungal growth on bread by volatile components from spices and herbs, and the possible application in active packaging, with special emphasis on mustard essential oil. Int. J. Food Microbiol..

[2] Jideani V.A., Vogt K. (2016). Antimicrobial packaging for extending the shelf life of bread—A review. Crit. Rev. Food Sci. Nutr..

[3] Saladino F., Manyes L., Luciano F.B., Mañes J., Fernandez-Franzon M., Meca G. (2016). Bioactive compounds from mustard flours for the control of patulin production in wheat tortillas. LWT-Food Sci. Technol..

[4] Deng Q., Zinoviadou K.G., Galanakis C.M., Orlien V., Grimi N., Vorobiev E., Lebovka N., Barba F.J. (2015). The effects of conventional and non-conventional processing on glucosinolates and its derived forms, isothiocyanates: Extraction, degradation, and applications. Food Eng. Rev..

[5] Cai J., Liu B., Su Q. (2001). Comparison of simultaneous distillation extraction and solid-phase microextraction for the determination of volatile flavor components. J. Chromatogr. A.

[6] Torrijos R., Righetti L., Cirlini M., Calani L., Mañes J., Meca G., Dall’Asta C. (2023). Phytochemical profiling of volatile and bioactive compounds in yellow mustard (*Sinapis alba*) and oriental mustard (*Brassica juncea*) seed flour and bran. LWT-Food Sci. Technol..

[7] Zhao D., Tang J., Ding X. (2007). Analysis of volatile components during potherb mustard (*Brassica juncea*, Coss.) pickle fermentation using SPME–GC–MS. LWT-Food Sci. Technol..

[8] Paunovic D.S., Solevic-Knudsen T., Krivokapic M., Zlatković B.P., Antic M. (2012). Sinalbin degradation products in mild yellow mustard paste. Hem. Ind..

[9] Wendlinger C., Hammann S., Vetter W. (2014). Various concentrations of erucic acid in mustard oil and mustard. Food Chem..

[10] Xiao Z.H., Niu L.Y., Liao X.J., Hu X.S. (2004). Determination of volatile flavor compounds in mustard oil, wasabi, and Chongcai leaf mustard by SPME/GC/MS. China Condiment.

[11] Ray A., Mohanty S., Jena S., Sahoo A., Acharya L., Panda P.C., Sial P., Duraisamy P., Nayak S. (2022). Drying methods affects physicochemical characteristics, essential oil yield and volatile composition of turmeric (*Curcuma longa* L.). J. Appl. Res. Med. Aromat. Plants.

[12] Bahmid N.A., Heising J., Dekker M. (2021). Multiresponse kinetic modelling of the formation, release, and degradation of allyl isothiocyanate from ground mustard seeds to improve active packaging. J. Food Eng..

[13] Gemert L.J. (2011). Odour thresholds. Compilations of Odour Threshold Values in Air, Water and Other Media.

[14] Lin X.H., Li R., Jiang Z.T. (2001). Study on the chemical composition in the volatile oil of Armoracia lapathifolia Gilib. Food Sci..

[15] Liu Y., Rossi M., Liang X., Zhang H., Zou L., Ong C.N. (2020). An integrated metabolomics study of glucosinolate metabolism in different brassicaceae genera. Metabolites.

[16] Pechacek R., Velisek J., Hrabcova H. (1997). Decomposition products of allyl isothiocyanate in aqueous solutions. J. Agric. Food Chem..

[17] Li Y.M., Huang J., Zhang Y., Liu Y.P. (2016). Extraction and analysis of volatile compounds in mustard oil by SPME-GC-MS. China Food Addit..

[18] Bell L., Kitsopanou E., Oloyede O.O., Lignou S. (2021). Important odorants of four brassicaceae species, and discrepancies between glucosinolate profiles and observed hydrolysis products. Foods.

[19] (2013). Chinese Food Safety Standard. Food Additive-Allyl isothiocyanate.

[20] (2014). Chinese Food Additive Use Standard.

[21] Li P., Zhao Y.M., Wang C., Zhu H.P. (2021). Antibacterial activity and main action pathway of benzyl isothiocyanate extracted from papaya seeds. J. Food Sci..

[22] Hossain M.S., Ye W., Hossain M.A., Okuma E., Uraji M., Nakamura Y., Mori I.C., Murata Y. (2013). Glucosinolate degradation products, isothiocyanates, nitriles, and thiocyanates, induce stomatal closure accompanied by peroxidase-mediated reactive oxygen species production in Arabidopsis thaliana. Biosci. Biotechnol. Biochem..

[23] Weerawatanakorn M., Wu J.C., Pan M.H., Ho C.T. (2015). Reactivity and stability of selected flavor compounds. J. Food Drug Anal..

[24] Li Y., Teng Z., Chen P., Song Y., Luo Y., Wang Q. (2015). Enhancement of aqueous stability of allyl isothiocyanate using nanoemulsions prepared by an emulsion inversion point method. J. Colloid Interface Sci..

[25] Jin F.Z., Ding R.X., Ding K., Han T., Chen X.N. (2020). Preparation of allyl isothiocyanate microencapsulation and its application in pork preservation. J. Food Process. Preserv..

[26] Chhajed S., Misra B.B., Tello N., Chen S. (2019). Chemodiversity of the glucosinolate-myrosinase system at the single cell type resolution. Front. Plant Sci..

[27] Li Y.M., Zhang Y., Huang J., Liu Y.P. (2016). The synthesis and odor characteristics of eight isothiocyanate flavor compounds. China Food Addit..

