# Supplementary material for: Comparative Analysis of Volatile Flavor Compounds in Seven Mustard Pastes via HS-SPME–GC–MS
Source: Molecules. 2023 Jul 18;28(14):5482. doi: 10.3390/molecules28145482 (PMC10383883; doi:10.3390/molecules28145482)
Supplement: Supplementary file 1 [file molecules-28-05482-s001.zip › molecules-2497958-supplementary.pdf]

Table S1. The relative concentration of volatile compounds in seven MP samples.

Compounds	Relative cconcentration (μg/g)							RI/RI* [b]	Qualitative method [d]
	MP 1	MP 2	MP 3	MP 4	MP 5	MP 6	MP 7		
Esters (9)									
Allyl isocyanate	2.63±0.19	1.86±0.08	5.43±1.02	- [a]	6.84±0.47	5.15±0.62	3.83±0.30	972/N [c]	MS
sec-Butylisothiocyanate-	3.29±0.75	4.38±1.19	-	8.31±1.58	3.27±0.35	-	-	1289/1287	MS, RI, S
Allyl isothiocyanate	2561.01±82.90	2496.43±77.48	3405.20±102.51	2540.38±42.03	6495.20±131.46	4734.59±67.05	3895.28±42.33	1363/1361	MS, RI, S
Allyl thiocyanate	520.50±38.56	499.57±27.09	714.63±51.27	524.18±49.98	1562.81±78.40	1092.93±71.03	867.37±58.63	1447/1440	MS, RI
3-Butenyl isothiocyanate	5.42±1.04	5.39±0.97	-	303.38±25.90	-	6.36±1.42	-	1451/1453	MS, RI
Diglycolmonoethylether acetate	2.00±0.21	2.34±0.03	6.10±0.10	-	-	-	-	1684/N	MS
3-(Methylthio)propyl isothiocyanate	-	1.62±0.32	-	2.55±0.17	-	-	-	1938/1979	MS, S
Benzyl isothiocyanate	1.96±0.05	2.20±0.04	1.22±0.00	2.70±0.31	1.60±0.08	1.31±0.09	-	2097/2107	MS, RI, S
Phenylethyl isothiocyanate	98.42±5.46	186.67±29.30	86.61±15.21	227.92±40.10	52.46±9.66	71.78±12.35	8.26±1.28	2227/2234	MS, RI, S
Sulfur-containing compounds (3)									
Allyl mercaptan	1.47±0.01	3.33±0.04	2.35±0.55	-	-	-	-	886/887	MS, RI, S
Diallyl sulfide	-	1.09±0.20	1.36±0.36	-	8.75±2.01	6.00±0.18	1.92±0.02	1148/1148	MS, RI, S
Diallyl disulphide	-	-	-	-	2.34±0.15	-	-	1477/1475	MS, RI, S
Nitriles (2)									
3-Butenenitrile	90.13±14.45	18.5±3.22	87.72±10.13	-	4.68±0.99	3.16±0.25	52.78±7.66	1176/1186	MS, RI
Benzenepropanenitrile	86.58±17.30	11.17±3.21	21.91±2.05	11.59±1.60	2.23±0.07	1.43±0.01	22.10±2.19	2035/2041	MS, RI
Ketones (3)									
α-Turmerone	4.47±1.51	-	-	-	-	-	-	2183/2245	MS
β-Turmerone	2.76±0.03	-	-	-	-	-	-	2250/N	MS
ar-Turmerone	3.49±0.10	-	-	-	-	-	-	2262/N	MS
Alkenes (3)									

α-Zingiberene	3.79±0.52	-	-	-	289.04±36.17	-	-	1718/1715	MS, RI
(-)-*β*-Sesquiphellandrene	4.20±0.96	-	-	-	-	-	-	1765/1765	MS, RI
Curcumene	2.33±0.12	-	-	-	-	-	-	1769/1768	MS, RI
Others (7)									
Carbon disulfide	28.22±4.32	143.4±11.60	136.91±18.94	16.37±0.92	184.56±24.22	88.93±8.40	39.42±5.14	730/733	MS, RI, S
Allyl chloride	8.16±1.98	9.40±2.00	20.00±3.34	3.11±0.41	9.16±0.39	5.33±0.72	7.41±1.63	806/814	MS, RI, S
Isothiazole	-	-	1.00±0.06	1.00±0.04	-	-	-	1214/N	MS
N-Allylacetamide	-	-	-	-	5.46±0.91	-	-	1761/N	MS
Hexanoic acid	-	1.71±0.01	-	3.41±0.06	-	-	3.30±0.26	1850/1850	MS, RI, S
3-Methylphenol	-	-	0.86±0.00	-	-	-	-	2087/2085	MS, RI, S
Dehydroacetic acid	-	-	-	-	-	31.90±0.80	-	2374/N	MS
Total	3430.83	3389.06	4491.3	3644.9	8628.4	6048.87	4901.67		

[a] "-": Not detected; [b] RI: retention index on a DB-WAX column, RI*: retention index in literature; [c] "N": retention index was not found in the literature; [d] "MS" means qualitative by mass spectrometry, "RI" means qualitative by retention index, and "S" means qualitative by the standard.

Table S2. Odor activity values of volatiles in seven samples.

Compounds	OT (μg/g) [a]	Odor description	OAV [b]						
			MP 1	MP 2	MP 3	MP 4	MP 5	MP 6	MP 7
sec-Butylisothiocyanate	0.09	Green	37	49	-[c]	92	36	-	-
Allyl isothiocyanate	0.049	Pungent mustard, horseradish	52266	50948	69494	51844	132555	96624	79495
3-Butenyl isothiocyanate	0.017	/	319	317	-	17846	-	374	-
Diglycolmonoethylether acetate	0.19	/	11	12	32	-	-	-	-
3-(Methylthio)propyl isothiocyanate	0.65	Earthy, vegetable, sulfurous, horseradish	-	2	-	4	-	-	-
Benzyl isothiocyanate	0.035	Dusty, medicinal, horseradish, oily	56	63	35	77	46	37	-
Phenylethyl isothiocyanate	0.24	Green, horseradish, gooseberry, watercress	410	778	361	950	219	299	34
Allyl mercaptan	0.018	Alliaceous, sulfurous, garlic, onion, roasted	82	185	131	-	-	-	-
Diallyl sulfide	0.1	Sulfurous, onion, garlic, horseradish, metallic	-	11	14	-	88	60	19
Diallyl disulphide	0.03	Alliaceous, onion, garlic, metallic	-	-	-	-	78	-	-
Benzenepropanenitrile	0.015	Powerful nasturtium	5772	745	1461	773	149	95	1473
Carbon disulfide	0.005	/	5644	28680	27382	3274	36912	17786	7884
Allyl chloride	18.6	/	<1	<1	1	<1	<1	<1	<1
Hexanoic acid	3	Sour, fatty, sweat, cheese	-	<1	-	1	-	-	1
3-Methylphenol	0.015	Medicinal, woody, leather, phenolic	-	-	57	-	-	-	-

[a] Odor detection threshold in water according to reference Gemert, L. J. (2011); [b] OAV is the ratio of the concentration to threshold; [c] "-": Not detected.
